# HER2 Amplification and Clinicopathological Characteristics in a Large Asian Cohort of Rare Mucinous Ovarian Cancer

**DOI:** 10.1371/journal.pone.0061565

**Published:** 2013-04-19

**Authors:** Wen-Yee Chay, Sung-Hock Chew, Whee-Sze Ong, Inny Busmanis, Xinyun Li, Sharyl Thung, Lynette Ngo, Sheow- Lei Lim, Yong-Kuei Lim, Yin-Nin Chia, Elisa Koh, Cindy Pang, Lay-Tin Soh, Jin Wang, Tew-Hong Ho, Sun-Kuie Tay, Soo-Kim Lim-Tan, Kiat-Hon Lim, John Whay-Kuang Chia, Liang-Kee Goh

**Affiliations:** 1 Department of Medical Oncology, National Cancer Centre, Singapore, Singapore; 2 Department of Pathology, KK Women and Children's' Hospital, Singapore, Singapore; 3 Division of Clinical Trials and Epidemiological Sciences, National Cancer Centre, Singapore, Singapore; 4 Department of Pathology, Singapore General Hospital, Singapore, Singapore; 5 School of Biological Sciences, Nanyang Technological University, Singapore, Singapore; 6 Department of Gynecological Oncology, KK Women and Children's' Hospital, Singapore, Singapore; 7 Department of Obstetrics and Gynecology, Singapore General Hospital, Singapore, Singapore; 8 Duke–National University of Singapore Graduate Medical School, Singapore, Singapore; 9 Saw Swee Hock School of Public Health, National University of Singapore, Singapore; University of Porto, Portugal

## Abstract

Mucinous epithelial ovarian cancer has a poor prognosis in the advanced stages and responds poorly to conventional chemotherapy. We aim to elucidate the clinicopathological factors and incidence of HER2 expression of this cancer in a large Asian retrospective cohort from Singapore. Of a total of 133 cases, the median age at diagnosis was 48.3 years (range, 15.8–89.0 years), comparatively younger than western cohorts. Most were Chinese (71%), followed by Malays (16%), others (9.0%), and Indians (5%). 24% were noted to have a significant family history of malignancy of which breast and gastrointestinal cancers the most prominent. Majority of the patients (80%) had stage I disease at diagnosis. Information on HER2 status was available in 113 cases (85%). Of these, 31 cases (27.4%) were HER2+, higher than 18.8% reported in western population. HER2 positivity appeared to be lower among Chinese and higher among Malays patients (p = 0.052). With the current standard of care, there was no discernible impact of HER2 status on overall survival. (HR = 1.79; 95% CI, 0.66–4.85; p = 0.249). On the other hand, positive family history of cancer, presence of lymphovascular invasion, and ovarian surface involvements were significantly associated with inferior overall survival on univariate and continued to be statistically significant after adjustment for stage. While these clinical factors identify high risk patients, it is promising that the finding of a high incidence of HER2 in our Asian population may allow development of a HER2 targeted therapy to improve the management of mucinous ovarian cancers.

## Introduction

Mucinous epithelial ovarian cancer (mEOC) accounts for 2% to 5% of all primary epithelial ovarian cancers (EOC). It is chemo- resistant [1], [2] and is associated with a poorer prognosis compared to other histological subtypes [3]. Information on optimal treatment is currently lacking. [4].

**Figure 1:**
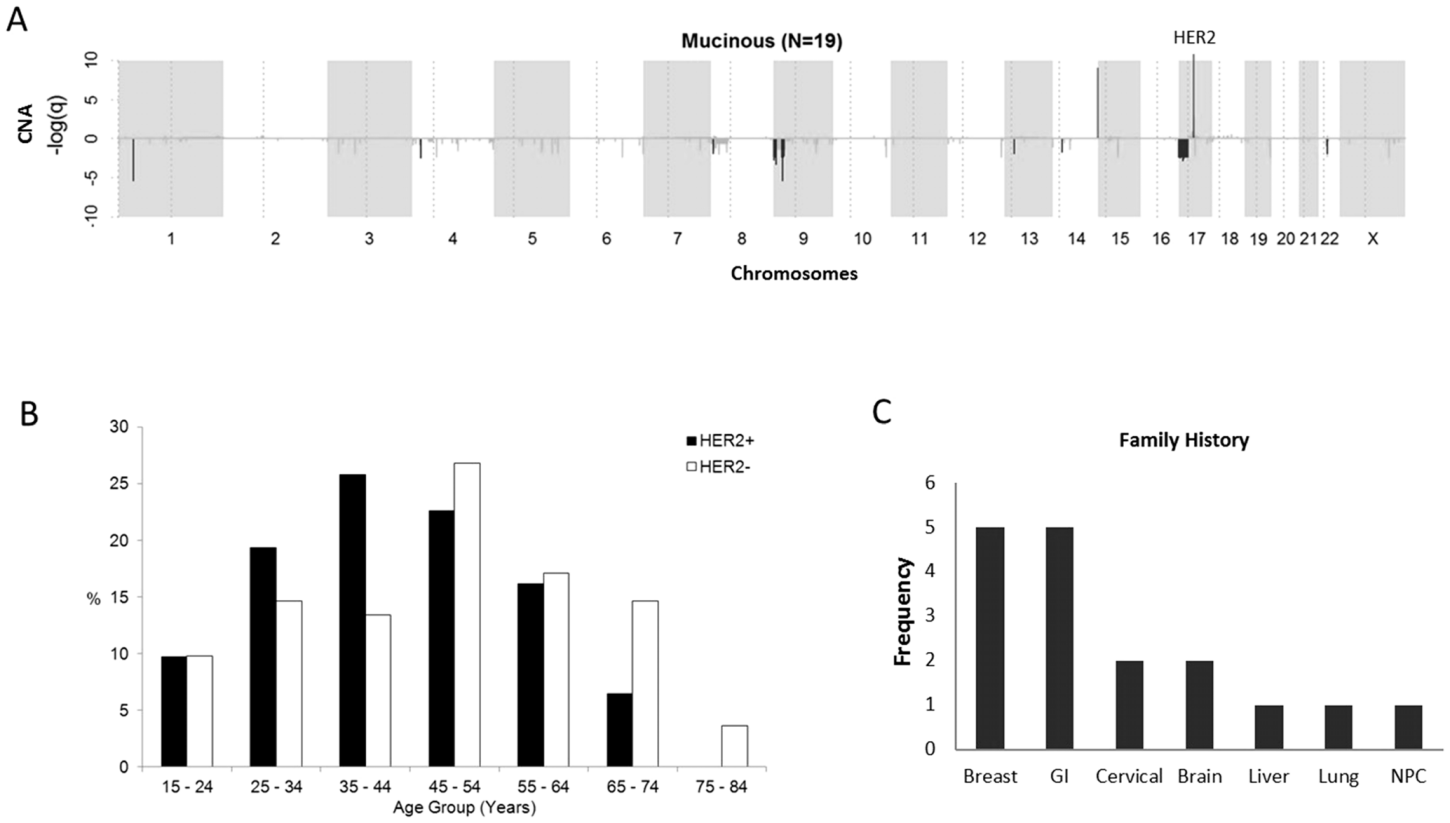
HER2 amplification, age, and frequency of cancers reported in family history of mEOC. (A) Previous genome-wide copy number alteration study on a small cohort of mEOC (n = 17) showed significant amplification of HER2. x-axis shows chromosomes 1-X, with alternating gray blocks. y-axis is the −log(q) where q is the false discovery rate. Positive values indicate amplification and negative values are deletion. (B) Age distribution was of normal distribution overall and for both HER2+ and HER2− cases. The median age was 48.3 (range: 15 to 89 years). (C) Frequency of reported cancers in family history. Majority of cancers were of breast and gastrointestinal (colon/stomach) origin. Note: some patients reported more than 1 case of cancer in family history.

HER2 has been found to be amplified in a significant number of mEOC, varying from 18.8% in a large western study (n = 154) [5] to 35.3% in a small Asian population (n = 17) [6]. Our previous study showed amongst the 4 major histopathology subtypes of EOC (i.e. clear cell, endometrioid, mucinous, and serous), mEOC harbored the highest prevalence of HER2 amplification [7]. Comparatively, mEOC do not show many copy number alterations except for a few focused regions including 9p21.3, and 17q12 which harbors HER2/ERBB2 (Figure 1a). Our study found deletion of HER2 in the other histotypes but not for mEOC [7]. HER2 is a member of the epidermal growth factor family of tyrosine kinase receptors involved in cellular proliferation and tumor cell metastasis. Amplification and over expression of HER2 has been shown in up to 15% of breast cancers and in 7–20% of gastric cancers. These cancers have been shown to carry a poorer prognosis compared to similar cancers of other histopathology subtypes. However, introduction of a monoclonal antibody (Trastuzumab) against the HER2 protein in combination with conventional chemotherapy has markedly improved response rates in HER2+ breast and gastric cancers. [8], [9], [10]

Given the rarity of mEOC, clinicopathological factors associated with clinical outcome of mEOC have been difficult to elucidate. In previous studies on Asian patients, the cohorts have been relatively small and clinicopathological factors were not well investigated. Hence we were motivated to: (i) ascertain HER2 status and its clinical relevance in a large cohort of mEOC samples of Asian descent; and (ii) investigate and identify novel clinicopathological factors that can improve identification of high risk patients.

## Methods

### Ethics

This study was approved by the institutional review boards (IRB) of the National Cancer Centre Singapore, KK Women's and Children's Hospital Singapore and Singapore General Hospital Singapore. IRB waiver of informed consent was approved as analyses were performed retrospectively on non-identifiable data (CIRB 2010/425/B).

### Case selection

The prospectively maintained gynaecologic oncology tumor databases were used to identify all patients with mEOCs. 199 cases of mucinous ovarian cancers were identified from 1963 to 2012. Tumour slides were retrieved and reviewed by 2 independent institutional gynaecologic pathologists and 133 cases dated between 1990 and 2012 which fulfilled the criteria of primary invasive mucinous ovarian cancer were selected. For the analysis, individual patient case notes were retrieved and data manually culled for age at diagnosis, ethnicity, Eastern Cooperative Oncology Group performance status, comorbidities (diabetes mellitus, hyperlipidemia, ischemic heart disease), tumor size, grade, serosal involvement, lymph node metastasis, type of surgery (hysterectomy, bilateral salpingooophorectomy, pelvic lymph node dissection, including fertility preservation), adjuvant chemotherapy, the date of initial diagnosis, recurrence and death. Death outcomes were supplemented by vital data obtained from the National Death Registry and tumor staging was determined using the 1998 FIGO criteria.

### Dual *in-situ* Hybridization

DISH of HER2 and chromosome 17 centromere probes were performed in an automated BenchMark ULTRA (Ventana Medical Systems, USA) slide stainer, using the INFORM HER2 Dual ISH DNA Probe Cocktail Assay (Ventana Medical Systems, USA) that allows detection of HER2 gene amplification by light microscopy. Tissue sections were deparaffinized and pretreated with Cell Conditioning 2 (CC2) at pH 6 at 86°C, and enzymatic digestion of proteins was performed with ISH protease 2 or 3 for variable lengths of time. Double-stranded DNA was denatured to allow hybridization of dinitrophenyl (DNP)-labeled HER2 DNA probes and digoxigenin (DIG)-labeled Chromosome 17 centromere probes. A stringency wash was performed at 72°C using sodium citrate, sodium chloride (SSC 10X) to wash off unbound or weakly bound probes. Detection of bound probes occurred separately, using the ultraView Silver ISH DNP and ultraView Red ISH DIG detection kits (Ventana Medical Systems, USA). Goat anti-mouse secondary antibodies conjugated to alkaline phosphatase (AP) were directed against primary DIG antibodies for detection of the centromere probes, whilst goat anti-rabbit secondary antibodies conjugated to horse radish peroxidase (HRP), were used against primary DNP antibodies.

### Immunohistochemistry

Immunohistocytochemistry (IHC) staining of HER2 protein was performed in a BenchMark ULTRA slide stainer utilizing the ultraView Universal DAB Detection kit (Ventana Medical Systems, USA). Tissue sections were deparaffinized and rehydrated with EZ Prep concentrate (10X) solution (Ventana Medical Systems, USA) and heat-mediated antigen retrieval was performed with cell conditioning 1 (CC1) (Ventana Medical Systems, USA) at 95°C. Slides were then treated with ultraView Di-aminobenzidene (DAB) inhibitor (Ventana Medical Systems, USA) and incubated with 100 µL of rabbit anti-HER2 monoclonal antibodies, immunoglobulin G (clone SP3) (ThermoScientific, USA) at a 1∶200 antibody diluent ratio for 24 minutes. Bound antibody was detected using ultraView DAB Detection Kit, where ultraView Horse Radish Peroxidase Multimer (anti-rabbit secondary antibody) (Ventana Medical Systems, USA) were added, followed by ultraView DAB H2O2 and chromogen (Ventana Medical Systems, USA).

### Scoring of DISH and IHC tests

#### DISH

The region of invasive carcinoma on tissue section was first marked out by a gynecological pathologist. Twenty non-overlapping nuclei were then enumerated on first count and the HER2/Chr17 ratio was then calculated. HER2 was considered amplified if the ratio was ≥2.2 and non-amplified if the ratio was <1.8 at this count. If the ratio fell between 1.8 and 2.2, an additional 20 nuclei were enumerated and the new ratio was calculated based on 40 nuclei. HER2 was amplified if the ratio on second count was ≥2.0, and non-amplified if <2.0.

#### IHC

The HER2 DAKO scoring system for gastric cancer [11] was adopted as a reference guide in our cohort of mEOC, due to the similarities in cell morphology, functionality and IHC staining patterns between the 2 tumor groups [12]. HER2 staining intensity and frequency were expressed as follows: IHC 0 (negative) – no staining or membrane staining in <10% of tumor cells; IHC 1+ (negative) – faint membrane staining in ≥10% of tumor cells and staining occurs only in part of the membrane; IHC 2+ (equivocal) – weak-to-moderate complete or basolateral membrane staining in ≥10% of tumor cells; IHC 3+ (positive) – moderate-to-strong complete or membranous membrane staining in ≥10% of tumor cells [11], [13]. Finally, correlations between HER2 gene copy number changes (DISH) and HER2 protein overexpression were performed.

HER2 positivity was defined as having IHC 3+, or IHC 2+ with DISH amplification. IHC 0, IHC 1+, or IHC 2+ with DISH non-amplification were considered HER2 negative.

### Statistical Analysis

To detect significant differences in the demographic and clinical characteristics between HER2+ and HER2− patients, categorical characteristics were compared using the Chi-square test or Fisher's exact test as appropriate. Mann-Whitney U test was used to compare continuous characteristics between the 2 groups. Overall survival (OS) duration was calculated from the date of diagnosis to the date of death. Progression-free survival (PFS) duration was calculated from the date of diagnosis to the date of first progression, relapse or death, whichever occurred first. Patients who did not develop any of these time-to-event endpoints were censored at their last follow-up date. The Kaplan-Meier method was used to estimate all survival distributions, the log-rank test was used to test the differences between survival curves and Cox proportional hazard models were fitted to estimate hazard ratios to assess the association of factors with each survival endpoint. The proportional hazards assumption underlying the Cox model was verified using Schoenfeld residuals, and a 2-sided p-value<0.05 was considered statistically significant. All analyses were performed using SAS version 9.3 (SAS Institute Inc., Cary, NC).

## Results

133 cases qualified as primary invasive mEOC and were included in this analysis. Clinical characteristics of the patients are summarized in Table 1. The median age of diagnosis for our study cohort was 48.3 years (range, 15.8–89.0 years), and the shape of the age-frequency curve was unimodal and symmetrical in distribution (Figure 1b). The majority of patients were Chinese (71%) and Malays and Indians constituted 16% and 5% of the cohort respectively. 24% of our mucinous cohort reported a significant family history of malignancy with breast, gastrointestinal, cervical and brain tumors the most frequent primary cancers cited (Figure 1c). In our study cohort, appendictomy was performed in 51% of patients and records of gastroscopy and colonscopy were available for 15% and 19% of subjects respectively.

**Table 1:** Clinicopathologic features of patients.

Characteristics	Categories	Patients (n = 133)
Median age at diagnosis, years (range)	-	48.3 (15.8–89.0)
Ethnic group	Chinese	94 (71%)
	Malays	21 (16%)
	Indians	6 (5%)
	Others	12 (9%)
Smoking history (n = 52)	Non-smoker/Ever-smoker	38 (73%)/14 (27%)
Family history of cancer (n = 59)	Negative/Positive	45 (76%)/14 (24%)
Presence of comorbidities (n = 126)	No/Yes	48 (38%)/78 (62%)
Stage at diagnosis (n = 125)	I	100 (80%)
	II	7 (6%)
	III	15 (12%)
	IV	3 (2%)
Tumour differentiation/grade (n = 117)	well	71 (61%)
	moderate	34 (29%)
	poor	12 (10%)
Tumour type (n = 130)	Mixed borderline/Non-borderline	50 (38%)/80 (62%)
Ovarian surface involvement (n = 122)	No/Yes	75 (61%)/47 (39%)
Lymphovascular invasion (n = 126)	No/Yes	118 (94%)/8 (6%)
Median CA125, *U/mL* (range) (n = 117)	-	71.3 (3.0–8812.5)
Received OGD (n = 126)	No/Yes	107 (85%)/19 (15%)
Received colonoscopy (n = 126)	No/Yes	102 (81%)/24 (19%)
Received appendectomy (n = 127)	No/Yes	62 (49%)/65 (51%)
Received omentectomy (n = 128)	No/Yes	10 (8%)/118 (92%)
Received chemotherapy (n = 124)	No/Yes	68 (55%)/56 (45%)
Received adjuvant chemotherapy (n = 123)	No/Yes	83 (67%)/40 (33%)

Abbreviation: OGD, oesophagogastroduodenoscopy.

Overall, the majority of patients (80.0%) with mucinous cancers were found to have stage I disease at diagnosis, with 6% of patient presenting in stage II and only 14% presented with advanced stage 3 and 4 diseases. Of the entire study cohort, 61% of mucinous tumors in our cohort were well differentiated tumors, 29% moderately differentiated and 10% poorly differentiated. 39% of cases had ovarian surface involvement (OSI) and lymphovascular invasion (LVI) was present in 6%.

### HER2 positivity and clinical factors

HER2 status was successfully ascertained in 113 cases. Of the 133 cases, 9 cases could not be assayed for IHC and 11 cases were IHC 2+ but unsuccessful in DISH. These 20 cases were filtered out in the HER2 status analyses. In summary, 31 samples or 27.4% (95% confidence interval 20.1% to 36.3%) were HER2+ and 82 were HER2−. Excellent concordance was observed between IHC and DISH assay for IHC 0, 1+, and 3+. Of the 16 cases that were IHC 2+, 4 had amplification ratio ≥2.0.

The observed proportion of HER2 positivity was higher in Malays than in Chinese in our cohort (Table 2). Although the proportion of Malays in our mucinous ovarian cancer cohort is similar to general Singapore population, there was a trend (marginal significance) towards more Malays in our study having HER2+ tumors than HER2− tumors (56% versus 44% respectively) as compared with the Chinese (23% vs 78%), Indians (17% vs 83%) and others (27% vs 83%) (p = 0.052). HER2− patients tend to present with an elevated CA 125 level as compared to HER2+ patients (p = 0.024). Marginal significance was observed in HER2− patients being older (p = 0.094), have non-borderline tumor type (p = 0.063) and presence of OSI (p = 0.075). Overall, there was no difference in smoking history, family history of cancer, stage at diagnosis, tumor grade and presence of lymphovascular invasion (LVI), between HER2+ and HER2− mucinous ovarian cancer groups (Table 2).

**Table 2:** Clinicopathologic features by HER2 status.

Characteristics	Categories	HER2+	HER2−	*P* value
Median age at diagnosis, years (range)	-	43.6 (17.6–74.9)	49.4 (15.8–82.1)	0.094
Ethnic group	Chinese (n = 80)	18 (23%)	62 (78%)	0.052
	Malays (n = 16)	9 (56%)	7 (44%)	
	Indians (n = 6)	1 (17%)	5 (83%)	
	Others (n = 11)	3 (27%)	8 (73%)	
Smoking history	Non-smoker (n = 29)	9 (31%)	20 (69%)	0.720
	Ever-smoker (n = 14)	3 (21%)	11 (79%)	
Family history of cancer	Negative (n = 39)	12 (31%)	27 (69%)	0.728
	Positive (n = 11)	4 (36%)	7 (64%)	
Presence of comorbidities	No (n = 40)	14 (35%)	26 (65%)	0.267
	Yes (n = 68)	17 (25%)	51 (75%)	
Stage at diagnosis	I (n = 88)	29 (33%)	59 (67%)	0.285
	II (n = 5)	0 (-)	5 (100%)	
	III (n = 11)	2 (18%)	9 (82%)	
	IV (n = 3)	0 (-)	3 (100%)	
Tumour differentiation/grade	well (n = 64)	19 (30%)	45 (70%)	0.717
	moderate (n = 28)	10 (36%)	18 (64%)	
	poor (n = 9)	2 (22%)	7 (78%)	
Tumour type	Mixed borderline (n = 45)	17 (38%)	28 (62%)	0.063
	Non-borderline (n = 65)	14 (22%)	51 (78%)	
Ovarian surface involvement	No (n = 66)	23 (35%)	43 (65%)	0.075
	Yes (n = 38)	7 (18%)	31 (82%)	
Lymphovascular invasion	No (n = 100)	29 (29%)	71 (71%)	0.186
	Yes (n = 7)	0 (-)	7 (100%)	
Median CA125, *U*/*mL* (range)	-	37.2 (6.1–415.7)	102.5 (3.0–8812.5)	**0.024**
Received OGD	No (n = 93)	29 (31%)	64 (69%)	0.148
	Yes (n = 16)	2 (13%)	14 (88%)	
Received colonoscopy	No (n = 90)	29 (32%)	61 (68%)	0.057
	Yes (n = 19)	2 (11%)	17 (89%)	
Received appendectomy	No (n = 51)	15 (29%)	36 (71%)	0.833
	Yes (n = 58)	16 (28%)	42 (72%)	
Received omentectomy	No (n = 7)	3 (43%)	4 (57%)	0.400
	Yes (n = 103)	28 (27%)	75 (73%)	
Received chemotherapy	No (n = 57)	18 (32%)	39 (68%)	0.419
	Yes (n = 49)	12 (24%)	37 (76%)	
Received adjuvant chemotherapy	No (n = 69)	19 (28%)	50 (72%)	0.745
	Yes (n = 36)	11 (31%)	25 (69%)	

Abbreviation: OGD, oesophagogastroduodenoscopy.

### Her 2 positivity and Survival

**Figure 2:**
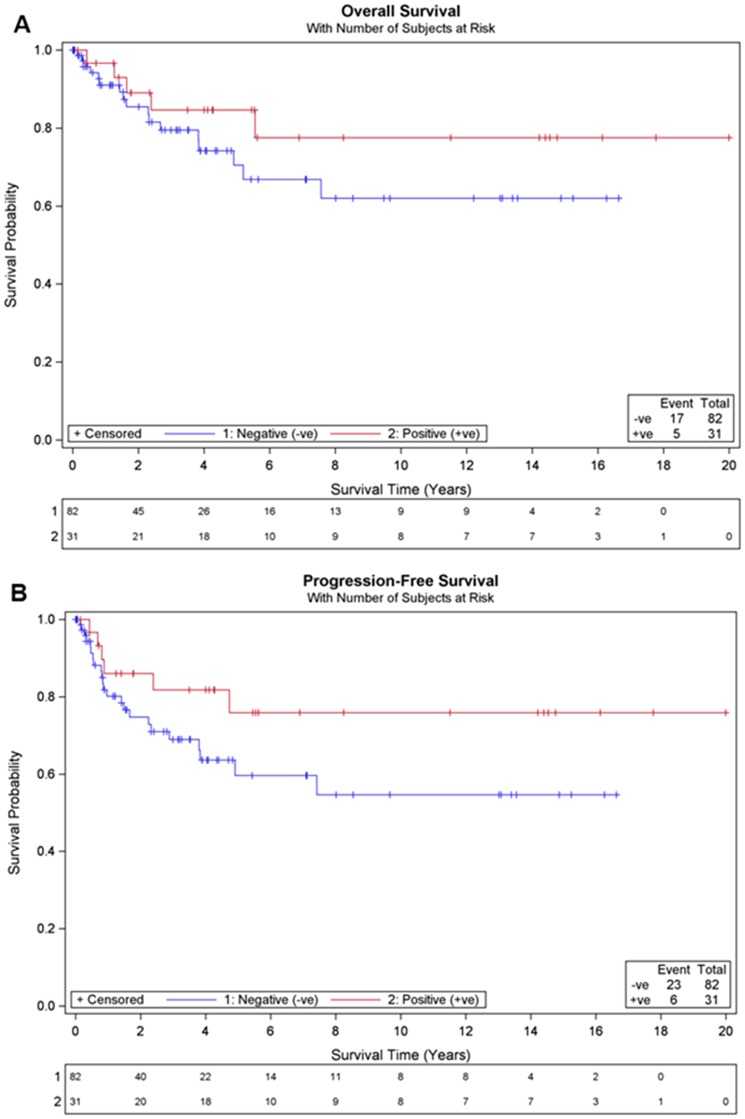
Survival outcomes by HER2 status. No statistical significance was observed for HER2+ compared to HER2− cases in (A) overall survival (log-rank p = 0.249), and (B) progression free survival (log-rank p = 0.120).

After a median follow-up of 2.8 years (range, 0–19.99 years), 29 recurrences and 22 deaths, the median overall survival (OS) was not reached for the study population and the 5-year OS rate was 75.4%. Overall, there was no statistically significant difference in OS between HER2+ and HER2− patients (p = 0.249) (Figure 2a). Similarly, HER2 status was not significant for PFS (p = 0.12) (Figure 2b). HER2− patients had shorter median follow-up than HER2+ patients (2.4 years vs 4.3 years; p = 0.012).There were more HER2− patients than HER2+ patients diagnosed in 2011 and 2012 (8.5% versus 3.2%), and a greater number of HER2− patients diagnosed before 2010 were lost to follow-up (23% vs 10% respectively). To assess the impact of inclusion of patients diagnosed in 2011 and 2012 on the survival outcomes in the study, a sensitivity analysis was conducted whereby the survival analyses by HER2 status were repeated based on patients diagnosed between 1990 and 2010. This sensitivity analysis did not lead to a different conclusion on the association of HER2 status with OS.

### Clinical Factors associated with Survival

Advanced age at diagnosis, positive family history of cancer, advanced disease stage at diagnosis, presence of LVI and ovarian surface involvement (OSI) were significantly associated with inferior OS on univariate analysis (Table 3). Tumor grade, ethnicity and smoking history did not appear to impact survival. Family history of cancer, LVI, and OSI continued to be statistically significant on multivariate analysis after adjustment for stage. As in OS, LVI and OSI, and in addition, receipt of chemotherapy were important factors determining the outcome of PFS in multivariate analysis after adjusting for stage (Table 4).

**Table 3:** Overall survival analysis.

Variable	Univariate Analysis		Stage-Adjusted Analysis	
	HR (95% CI)	*P* value^a^	HR (95% CI)	*P* value^b^
Age at diagnosis (per year increase)	1.03 (1.01–1.06)	**0.011**	1.02 (0.99–1.05)	0.191
Ethnic group (Malays vs Chinese)	0.39 (0.12–1.27)	0.127	0.68 (0.20–2.33)	0.943
Ethnic group (Indians vs Chinese)	0.00 (NE)		0.00 (NE)	
Ethnic group (Others vs Chinese)	0.00 (NE)		0.00 (NE)	
Smoking history (Ever-smoker vs non-smoker)	0.98 (0.20–4.91)	0.980	0.47 (0.06–3.45)^c^	0.458
Family history of cancer (Positive vs Negative)	5.88 (1.40–24.79)	**0.006**	7.95 (1.30–48.65)^c^	**0.025**
Presence of comorbidities (Yes vs No)	1.16 (0.55–2.42)	0.694	0.75 (0.34–1.65)	0.475
Stage at diagnosis (II vs I)	4.85 (1.57–14.97)	**<0.001**	-	-
Stage at diagnosis (III vs I)	15.42 (6.54–36.36)		-	
Stage at diagnosis (IV vs I)	28.51 (7.38–110.22)		-	
Tumour differentiation/grade (moderate vs well)	1.15 (0.48–2.78)	0.093	1.15 (0.47–2.82)	0.848
Tumour differentiation/grade (poor vs well)	2.77 (1.05–7.29)		1.35 (0.46–3.95)	
Tumour type (Non-borderline vs mixed borderline)	1.75 (0.81–3.80)	0.153	1.33 (0.58–3.07)	0.500
Ovarian surface involvement (Yes vs No)	7.80 (3.14–19.35)	**<0.001**	4.14 (1.45–11.80)	**0.008**
Lymphovascular invasion (Yes vs No)	10.25 (4.39–23.92)	**<0.001**	5.58 (2.05–15.21)	**0.001**
CA125 (per *U*/*mL* increase)	1.00 (1.00–1.00)	0.916	1.00 (1.00–1.00)	0.461
Received chemotherapy (Yes vs No)	4.26 (1.83–9.89)	**<0.001**	1.66 (0.60–4.60)^d^	0.335
Received adjuvant chemotherapy (Yes vs No)	1.18 (0.58–2.41)	0.646	1.01 (0.43–2.38)	0.974
HER2 status (HER2− vs HER2+)	1.79 (0.66–4.85)	0.249	1.01 (0.34–2.97)	0.988
*Among HER2+ patients:* HER2 amplification ratio (per unit increase)	0.75 (0.49–1.15)	0.185	0.72 (0.44–1.18)^c^	0.193

Abbreviation: HR, hazard ratio; CI, confidence interval; NE, not estimable.

a *P* values for age at diagnosis, CA125 and HER2 amplification ratio were based on Wald test, and *P* values for all other variables were based on the log-rank test.

b Based on Wald test.

c To interpret with caution as there were <10 deaths in the fitted multivariable model.

d Departures from proportionality assumption. The time-varying effects of receipt of chemotherapy were further accounted for by including a time-by-covariate interaction term in the Cox model. Based on the extended model, there was no significant association between OS and chemotherapy.

**Table 4:** Progression-free survival analysis.

Variable	Univariate Analysis		Stage-Adjusted Analysis	
	HR (95% CI)	*P* value^a^	HR (95% CI)	*P* value^b^
Age at diagnosis (per year increase)	1.02 (0.99–1.04)	0.057	1.01 (0.99–1.04)	0.287
Ethnic group (Malays vs Chinese)	0.43 (0.15–1.21)	0.167	0.70 (0.24–2.06)	0.731
Ethnic group (Indians vs Chinese)	0.37 (0.05–2.70)		0.37 (0.05–2.76)	
Ethnic group (Others vs Chinese)	0.00 (NE)		0.00 (NE)	
Smoking history (Ever-smoker vs non-smoker)	2.09 (0.66–6.58)	0.199	0.81 (0.16–4.04)	0.797
Family history of cancer (Positive vs Negative)	2.22 (0.72–6.86)	0.154	2.91 (0.75–11.26)	0.121
Presence of comorbidities (Yes vs No)	1.19 (0.61–2.34)	0.605	0.91 (0.44–1.87)	0.799
Stage at diagnosis (II vs I)	3.88 (1.30–11.52)	**<0.001**	-	-
Stage at diagnosis (III vs I)	10.45 (4.80–22.71)		-	
Stage at diagnosis (IV vs I)	16.43 (4.54–59.51)		-	
Tumour differentiation/grade (moderate vs well)	1.24 (0.54–2.88)	**0.001**	1.20 (0.52–2.78)	0.224
Tumour differentiation/grade (poor vs well)	4.33 (1.86–10.07)		2.25 (0.89–5.67)	
Tumour type (Non-borderline vs mixed borderline)	1.91 (0.95–3.84)	0.065	1.33 (0.63–2.80)	0.447
Ovarian surface involvement (Yes vs No)	8.86 (3.82–20.53)	**<0.001**	5.26 (2.04–13.58)	**0.001**
Lymphovascular invasion (Yes vs No)	8.13 (3.59–18.42)	**<0.001**	4.60 (1.81–11.71)	**0.001**
CA125 (per *U*/*mL* increase)	1.00 (1.00–1.00)	0.617	1.00 (1.00–1.00)	0.803
Received chemotherapy (Yes vs No)	5.92 (2.60–13.50)	**<0.001**	3.01 (1.18–7.68)	**0.021**
Received adjuvant chemotherapy (Yes vs No)	1.69 (0.89–3.23)	0.106	1.63 (0.76–3.49)	0.212
HER2 status (HER2− vs HER2+)	2.02 (0.82–4.96)	0.120	1.30 (0.50–3.38)	0.586
*Among HER2+ patients:* HER2 amplification ratio (per unit increase)	0.72 (0.48–1.08)	0.109	0.69 (0.43–1.09)^c^	0.111

Abbreviation: HR, hazard ratio; CI, confidence interval; NE, not estimable.

a P values for age at diagnosis, CA125 and HER2 amplification ratio were based on Wald test, and P values for all other variables were based on the log-rank test.

b Based on Wald test.

c To interpret with caution as there were <10 deaths in the fitted multivariable model.

## Discussion

Ovarian mucinous tumors tend to have a poor prognosis in advanced stages and response to chemotherapy is generally poor in comparison to other histology subtypes of ovarian cancer. There is mounting evidence that distinct mutations and genomic aberrations exist in each histological subtype of ovarian cancers, suggesting that treatment of ovarian cancer could be stratified according to histology subtypes.

The current study reports on HER2 status and clinicopathological factors in the largest cohort of mEOC in an Asian setting. It carries three significant implications. Firstly, compared to a recent report by Anglesio et al that the incidence of HER2 positivity was 18.8% (n = 154) in the western populations [5], a higher HER2 overexpression rate of 27.4% (95% confidence interval 20.1% to 36.3%) was found in this current study. Although a previous local study limited to a sample size of 17 patients only had reported a HER2 positivity rate of 35%, the rate falls within the 95% confidence interval of the current study. [6]. Our findings further demonstrated that there was a higher incidence of HER2+ in Malays (56%) compared to Chinese (23%) and Indians (17%). We believe that there is an ethnic difference in genetic makeup for mucinous ovarian cancers which may also account for the reported difference in the incidence of HER2 positivity between Asians and Caucasians.

Secondly, we found that 80.0% of our mEOC patients had stage I disease at diagnosis as compared to 55% to 60% in reports from the western populations.[5], [14]. Our data also showed that clinicopathological features in terms of smoking history, family history of cancer, stage at diagnosis, tumor grade, and presence of LVI were similar between HER2+ and HER2− patients. Our study revealed that factors associated with a poorer survival include the presence of family history, ovarian surface involvement and lymphovascular involvement by tumor. The presence of ovarian surface involvement affects the overall survival significantly (HR of 4.14) in mucinous ovarian cancers. In comparison, ovarian surface involvement has not yet been found to be a prognostic factor for overall survival in other histotypes such as serous ovarian cancer. A possible reason is mucinous ovarian cancers responds poorly to chemotherapy as compared to serous subtypes. Family history was associated with a worse prognosis even after adjustment for stage at diagnosis (HR = 7.95). Of interest, the comparable incidence of gastrointestinal cancers with breast cancer (which is a known associative risk factor in family history) and the notion that mucinous ovarian cancer is often associated pathologically with intestinal cancer suggest that genetics in gastrointestinal cancers may also contribute to ovarian cancer. Overall, these factors allow us to better identify at risk individuals and institute early interventions. History of smoking has been reported to be a risk factor for mucinous epithelial ovarian cancers and no other histotypes of epithelial ovarian cancer [15]. In this study, no significance was observed for smoking status with overall survival (p = 0.72).

Thirdly, contrary to the report from McCaughan et al who found HER2+ ovarian cancer patients to have a poor survival rate [16], HER2 status did not show an impact on overall survival rate in the current cohort. Our finding is similar to the results reported in the Western population by Anglesio et al. However, one has to note that McCaughan's report was based on HER2 status in all histology subtypes while the current compared the impact of HER2 status among mEOC alone. The general poor chemo-responsiveness of mEOC could have masked the importance of HER2. This opens the opportunity to investigate if inclusion of targeted therapy to HER2 receptor to conventional chemotherapy would make a difference to the survival rate of mEOC. Experience can be drawn from management of other cancers. For example, McAlpine et al investigated the use of trastuzumab in combination with conventional chemotherapy in 3 patients and of which one patient responded dramatically to the use of Trastuzumab [12]. Recent trial on combination of Pertuzumab, Trastuzumab and Docetaxel showed improved outcome for patients with HER2+ metastatic breast cancer [17]. Further studies are needed to further evaluate prospectively the use of HER2 inhibitors in the treatment of epithelial ovarian cancers.

Angelsio et al also showed that HER2+ and KRAS mutations (i.e. KRAS+) are almost mutually exclusive in mucinous ovarian cancers. Interestingly, a double negative subtype (i.e. HER2− and KRAS−) showed poorer prognosis, similar to the poorer prognosis observed for triple-negative subtype in breast cancer [18], [19]. Further work is needed to determine the double-negative mucinous ovarian cancer subtype in our population. Determining the nature and frequency of these activating mutations will eventually allow us to better individualize treatment for our patients.

Although our study is the largest Asian mucinous ovarian cancer cohort to date, there are limitations in the study. In our cohort, differences in follow up duration between HER2+ and HER2− patients may have affected the survival outcomes, although we have taken the steps to assess the sensitivity due to the difference. The sensitivity analyses did not show significance. The role of tumor intra-heterogeneity may also affect HER2 expression [20]. In view of this, we have employed whole tissue sections in the ascertainment of HER2 status in this study, instead of using tissue microarray as in other studies. Together with independent review by 2 pathologists in each institute, this should improve robustness in the determination of HER2 positivity.

## Conclusion

This study is the largest cohort to report clinicopathological factors and the incidence of HER2 mucinous ovarian cancer in an Asian setting. The data shed light on the differences in HER2 prevalence between Asian and western cohorts, and within our Asian cohort, between ethnic subpopulations. The high prevalence of HER2 in mEOC suggests the potential for HER2 targeted treatment in this relatively chemo-resistant and rare cancer.
